# Giant Pancreatic Lipoma of the Uncinate Process: A Case Report

**DOI:** 10.7759/cureus.114746

**Published:** 2026-08-18

**Authors:** Iván L Rosso, Angela Duque Jimenez, Hector F Beltrán, Gina T Sanchez, Wilmer Aponte

**Affiliations:** 1 Radiology, Universidad Nacional de Colombia, Bogotá, COL

**Keywords:** lipoma, liposarcoma, pancreas, pancreatic neoplasms, retroperitoneal neoplasms

## Abstract

Lipoma is a common benign tumor composed of mature adipose tissue; however, its occurrence in the pancreatic parenchyma is rare. It is usually identified as an incidental finding on imaging studies ordered for other reasons. We present the case of a 58-year-old male patient who underwent evaluation for a left renal mass, in whom a giant pancreatic lipoma (>5 cm) was detected by contrast-enhanced abdominal computed tomography (CT) and subsequently characterized with magnetic resonance imaging (MRI). Given the rarity of this condition and, in this case, its large size, rigorous imaging characterization is essential to differentiate it from malignant neoplasms and guide appropriate therapeutic management.

## Introduction

Pancreatic neoplasms present a constant diagnostic challenge; the vast majority are of epithelial origin and are typically malignant. However, tumors of mesenchymal origin are extremely rare, and among them, pancreatic lipoma stands out for its low incidence, estimated at less than 0.1% of pancreatic tumors [1]. It is characterized as a benign lesion composed of well-encapsulated mature adipose tissue. Its diagnosis is often an incidental finding in studies performed for other reasons, due to the widespread use of advanced imaging techniques such as computed tomography (CT) and magnetic resonance imaging (MRI) [2]. The fundamental value of reporting these cases lies in the ability to differentiate this entity from malignant processes, thereby avoiding unnecessary invasive surgical interventions in favor of conservative management and clinical surveillance [3]. We present the case of a large pancreatic lipoma in the uncinate process, characterized by MRI in a patient with a concomitant renal tumor, with the intention of showing how the use of other imaging features besides size allows for an accurate diagnosis of this pathology.

## Case presentation

**Figure 1:**
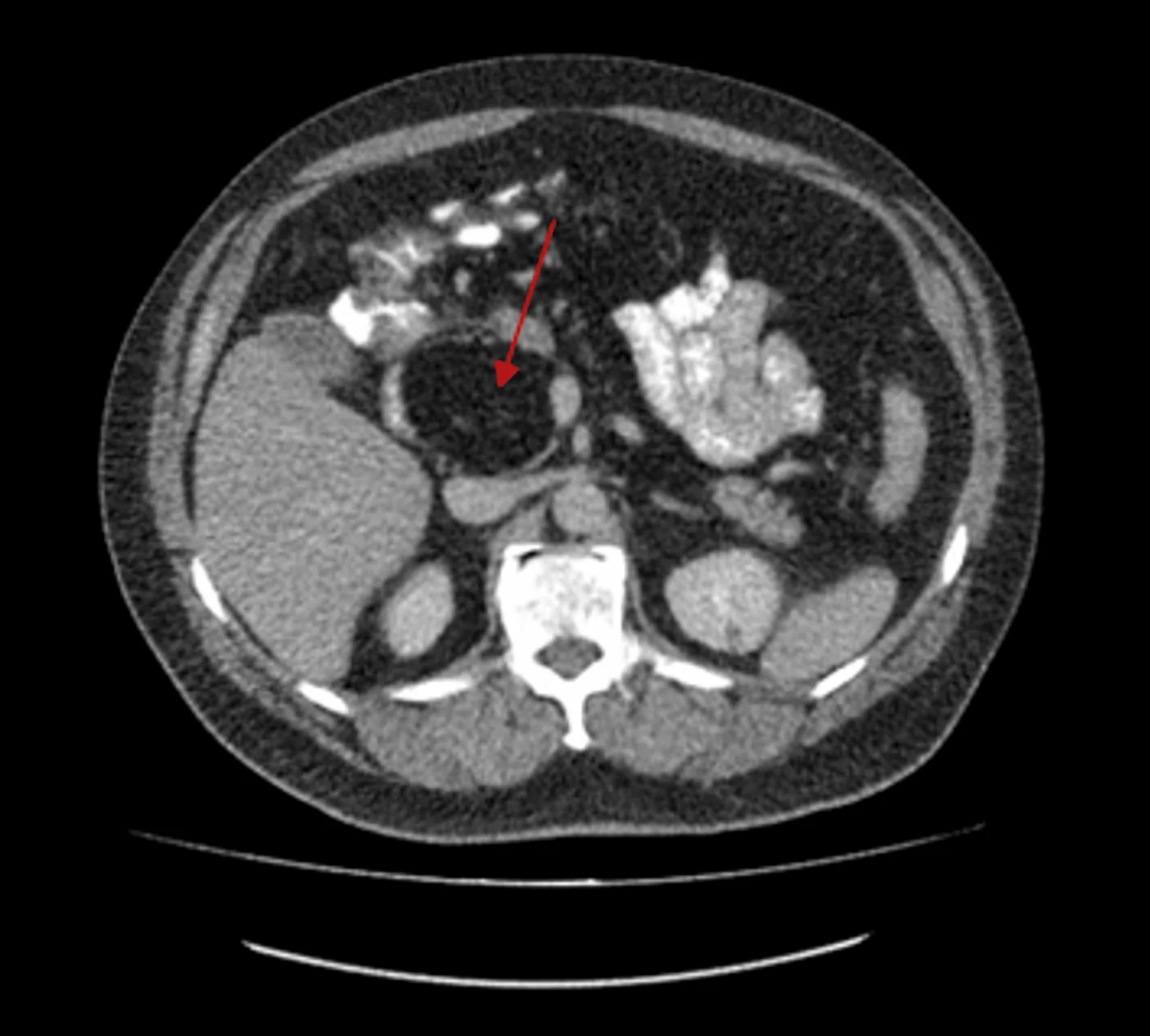
Contrast-enhanced abdominal computed tomography scan (axial view) A rounded lesion involving the pancreatic head and uncinate process, with well-defined margins and homogeneous fat attenuation (-60 HU), shows no contrast enhancement and displaces adjacent structures without evidence of infiltration (red arrow).

A 58-year-old male patient presented with mild, irritative urinary symptoms. An abdominal CT scan (Figure 1) was performed and revealed a solid lesion in the interpolar region of the left kidney. Incidentally, a 6-cm well-circumscribed hypodense lesion was identified in the pancreatic head and uncinate process, with attenuation values consistent with fatty tissue. Laboratory and paraclinical findings were within normal ranges (Table 1).

**Table 1:** Laboratory results

Laboratory	Result	Normal reference range	Unit
White blood cell count	8,400	4,000-11,000	cells/mm^3^
Hemoglobin count	15	13.5-17.5	g/dL
Platelet count	272,000	150,000-400,000	cells/mm^3^
Urinalysis			
Erythrocytes in urine	3	0-4	cells/µL
Nitrites	Negative	Negative	-
Leucocytes in urine	3	0-5	cells/µL
Serum creatinine	0.98	0.74-1.35	mg/dL
Prothrombin time (PT)	12.4	11-13.5	seconds
Partial thromboplastin time (PTT)	30	25-35	seconds
International normalized ratio (INR)	0.9	0.8-1.1	-
Serum amylase	53	30-110	U/L
Serum lipase	62	0-160	U/L
Total bilirubin	1.1	0.1-1.2	mg/dL
Direct bilirubin	0.2	<0.3	mg/dL
Indirect bilirubin	1.08	0.1-1.0	mg/dL
Alanine aminotransferase (ALT)/glutamate pyruvate transaminase (GPT)	26	7-55	U/L
Aspartate aminotransferase (AST)/glutamate oxaloacetate transaminase (GOT)	22	8-33	U/L
Alkaline phosphatase	58	44-147	U/L
Carbohydrate antigen (CA) 19-9	12	<37	U/mL
Carcinoembryonic antigen (CEA)	1.3	<3.0	ng/mL

**Figure 2:**
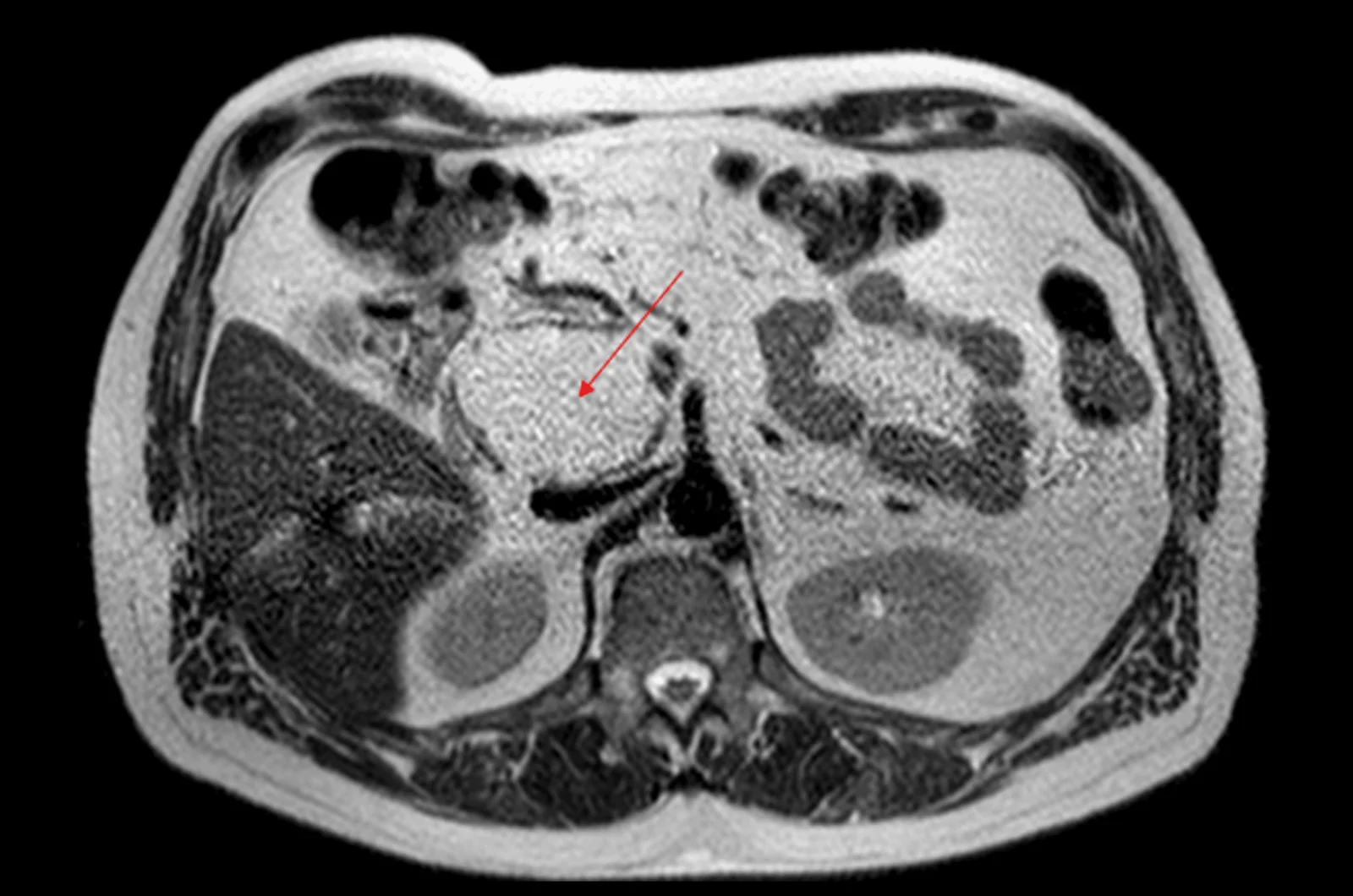
Contrast-enhanced abdominal magnetic resonance imaging, T2-weighted sequence (axial plane) A well-defined, hyperintense, rounded lesion is seen in the uncinate process of the pancreas, in close proximity to the splenoportal confluence and the portal vein (red arrow).

**Figure 3:**
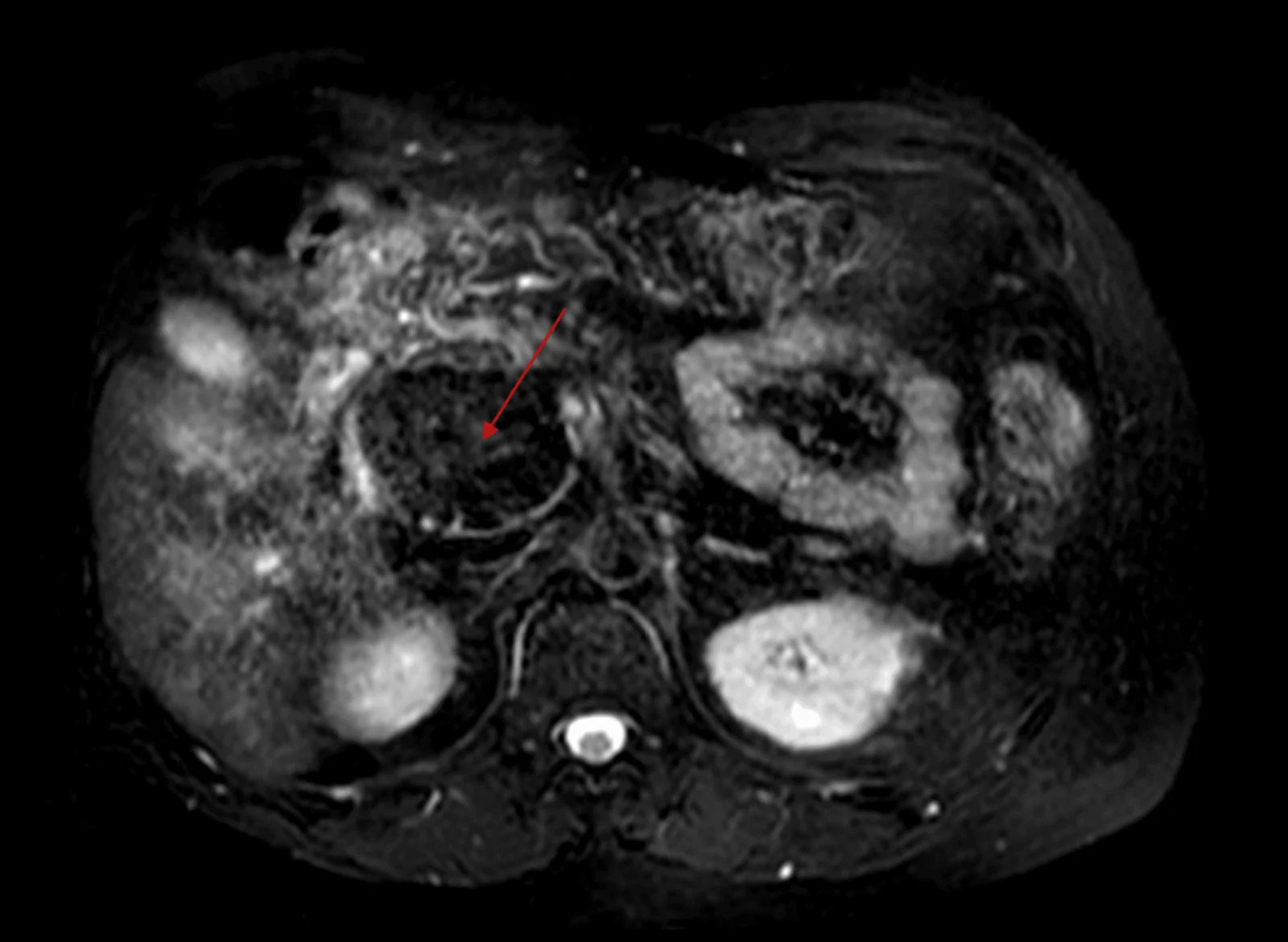
Contrast-enhanced abdominal magnetic resonance imaging, T2-weighted sequence with fat saturation (axial plane) The lesion shows homogeneous and complete loss of signal on fat-suppressed sequences (red arrow).

**Figure 4:**
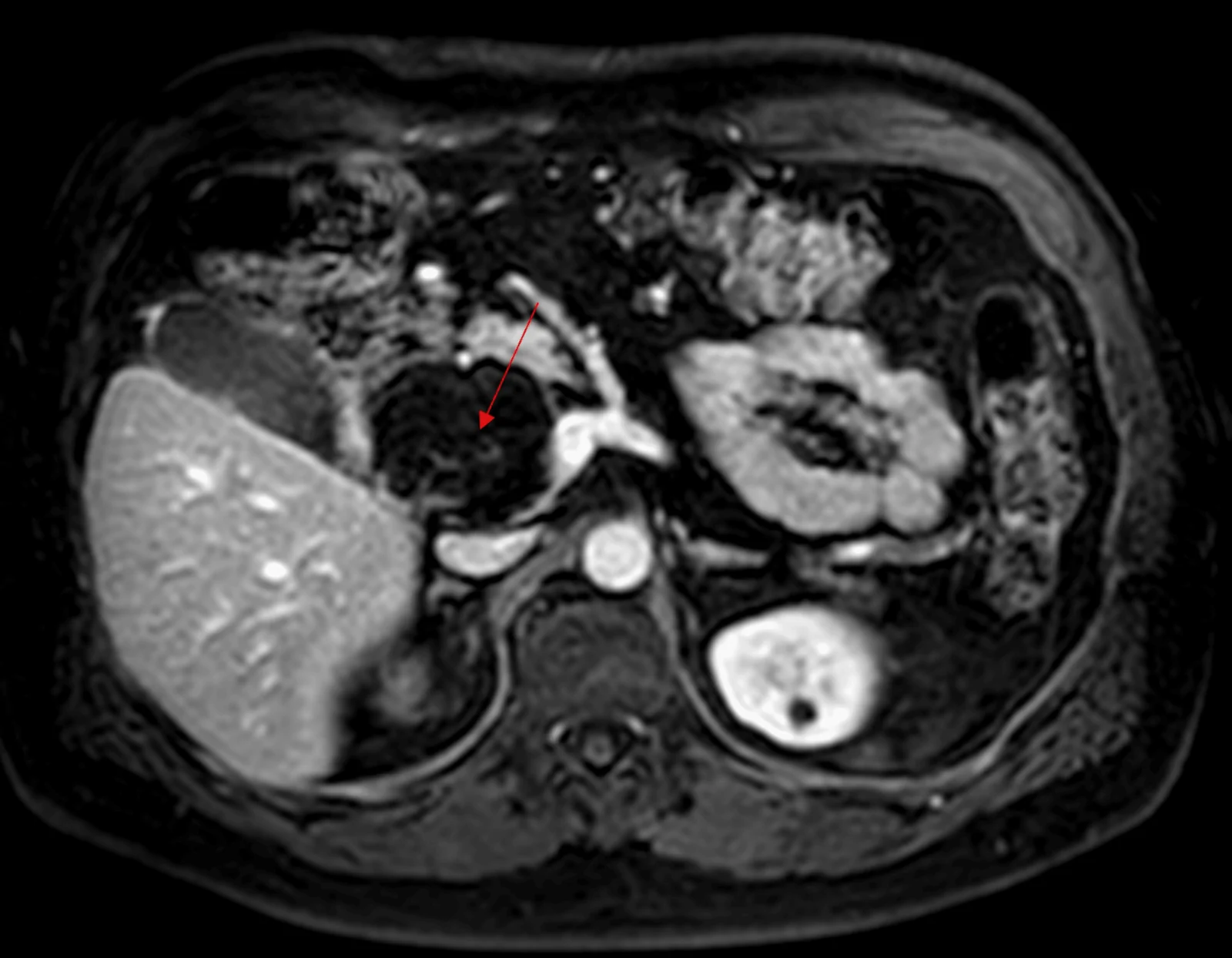
Contrast-enhanced abdominal magnetic resonance imaging, T1-weighted sequence in the portal phase (axial view) The lesion does not enhance with contrast and does not invade adjacent structures (red arrow).

**Figure 5:**
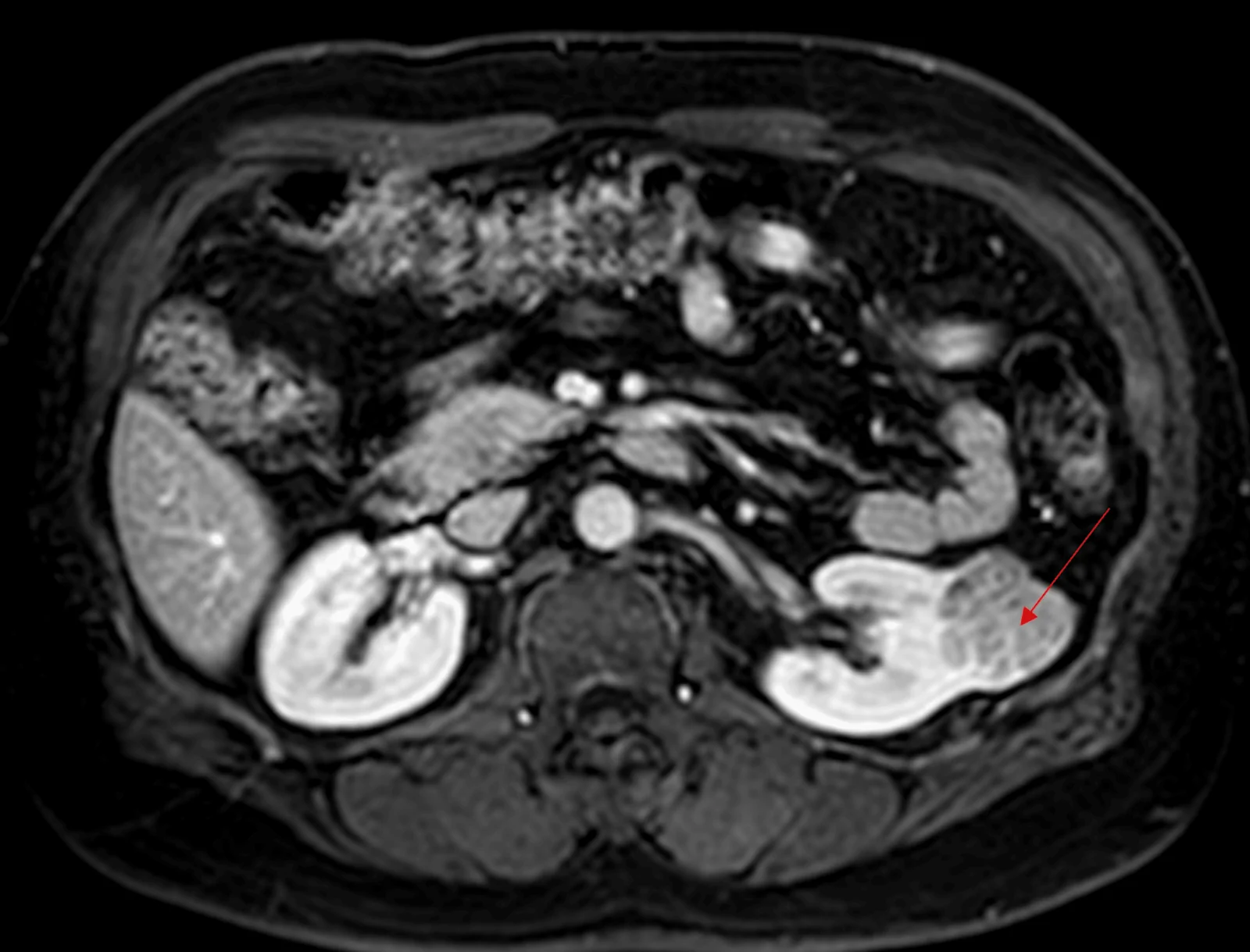
Contrast-enhanced abdominal magnetic resonance imaging, T1-weighted sequence in the portal phase (axial view) An eccentric, heterogeneous renal lesion in the interpolar region of the left kidney with heterogeneous enhancement in the portal phase has a Clear Cell Likelihood Score (ccLS) of 3, indicating intermediate probability for clear cell carcinoma (red arrow).

Contrast-enhanced abdominal MRI showed that the mass exhibited high signal intensity on T1- and T2-weighted sequences (Figures 2-3), with complete signal loss on fat-suppressed sequences (Figure 4). There was no diffusion restriction, and no enhancement was demonstrated on contrast-enhanced sequences. The mass displaced the second portion of the duodenum, the common bile duct, and the splenoportal confluence, with no evidence of tissue infiltration or obstructive involvement of adjacent vascular structures. Additionally, a mass was identified in the left kidney with a Clear Cell Likelihood Score (ccLS) of 3, indicating an intermediate probability of clear cell renal cell carcinoma (ccRCC) (Figure 5). It should be noted that despite the findings, the patient did not show any gastrointestinal symptoms.

Regarding the renal mass, at three months, surgical management was performed with a left partial nephrectomy; histopathological examination confirmed the diagnosis of ccRCC. Following the procedure, a new abdominal CT scan was performed, which showed that the pancreatic lesion remained stable, with no significant changes.

## Discussion

A pancreatic lipoma is a benign neoplasm of mesenchymal origin that rarely affects this organ, with an incidence of 0.012% in the general population. It is a tumor composed of mature adipose tissue at its core, with the occasional presence of thin connective tissue septa, bounded by a thin fibrous capsule that separates it from the rest of the pancreatic parenchyma. The first case was reported in 1989 by Bigard et al. as an ultrasound finding [4]. It is currently hypothesized that its origin is due to an anomalous fusion between the ventral and dorsal pancreatic buds during the embryonic stage, where a portion of retroperitoneal or mesenteric fat becomes trapped when these buds join at the head or the uncinate process of the pancreas, which are the most common locations [5]. The pathophysiology of their formation when located in the tail or body remains unknown. According to Xiao et al., by 2019, 169 cases had been reported in the literature, of which only 22 were confirmed by pathology. Of these, 87 occurred in men and 75 in women, with a predominance of middle-aged and older adult patients. The most common location was the head (n=70 cases), and most lesions measured less than 5 cm (n=150 cases) [6].

It is often identified as an incidental finding during the evaluation of other conditions and, in most cases, is asymptomatic. However, in lesions larger than 5 cm, symptoms such as abdominal pain, a sense of a mass, jaundice, and even portal hypertension may occur as a result of the compression of adjacent structures [5].

Contrast-enhanced abdominal CT is the most commonly used diagnostic method, allowing for a definitive diagnosis in up to 95.6% of cases, according to Zhan et al. [5]. The described criteria include a solid, homogeneous, well-defined mass without continuity with the peripancreatic adipose tissue; absence of invasion of adjacent organs; presence of a thin, continuous capsule; density values between -30 and -150 HU; and absence of enhancement following contrast administration [2]. In this patient's case, it is worth noting that although the lesion measures more than 5 cm, it has a homogeneous density of -60 HU (consistent with fatty tissue), well-defined borders, no enhancement, no septa, and no signs suggestive of infiltration of adjacent organs. Based on all of the above, it can be concluded in this case that the mass is benign despite its size.

It is also necessary to clarify that certain genetic disorders that could explain the concurrent occurrence of the renal mass and the pancreatic lipoma were ruled out in this patient, including the following: First is tuberous sclerosis. The patient did not present with skin abnormalities, neurological involvement such as epilepsy, or neurodevelopmental abnormalities. Likewise, he had no history of other renal masses such as angiomyolipomas. Second is von Hippel-Lindau disease. The patient had no history of tumors in other organs nor a family history of this condition. Third is systemic lipomatosis. The patient reported no history of lipomas elsewhere in the body. Imaging studies did not identify lipomas in other organs.

In some cases, it may be necessary to supplement the evaluation with MRI, in which the lesion is typically characterized as homogeneous and hyperintense on T1- and T2-weighted sequences, with a signal similar to that of subcutaneous fat, and with signal suppression on short-time inversion recovery (STIR) sequences and fat-saturation (Fat-Sat) techniques [3].

Finally, in cases of diagnostic uncertainty or when malignancy is highly suspected, the literature reports the use of non-routine studies such as positron emission tomography (PET)/CT, in which fluorine-18 fluorodeoxyglucose (18F-FDG) uptake is typically not evident, as well as biliopancreatic endoscopic ultrasound, which allows for better morphological characterization and the collection of samples via fine-needle aspiration biopsy [5].

The main differential diagnoses to consider for this condition include the following: First is focal fatty replacement. This is characterized by benign infiltration of peripancreatic fat into the parenchyma; there is no clear boundary between the mass and the peripancreatic fat, nor is a capsule evident [2,5]. It may show areas of internal enhancement with contrast administration due to the presence of portions of normal tissue within the area of replacement [2]. Histopathologically, foci of atrophic parenchyma associated with adipose tissue are observed [5].

Second is lipomatous pseudohypertrophy of the pancreas or diffuse lipomatosis of the pancreas. This condition is associated with a gradual and progressive diffuse replacement of exocrine tissue by fat, leading to an enlargement of the pancreas. It typically occurs in patients with cystic fibrosis or in certain congenital disorders such as Shwachman-Diamond syndrome or Johanson-Blizzard syndrome. It typically shows areas of focal enhancement on CT scans due to the presence of islands of normal parenchyma trapped within the replacement fatty parenchyma [2].

Third is liposarcoma. It is typically a heterogeneous mass with poorly defined, irregular borders, containing thickened septa and internal calcifications, which infiltrates the adjacent parenchyma, is larger in size (>5 cm), and enhances with contrast medium [4] (Table 2). When they exhibit a high degree of differentiation, they are often difficult to characterize on imaging studies, and a biopsy is required to confirm the diagnosis [6].

**Table 2:** Comparative table of diagnostic imaging features for lipoma and liposarcoma

Characteristics	Lipoma	Liposarcoma
Margins	Smooth, well-defined	Irregular
Size	≤5 cm	>5 cm
Density	Homogeneous tissue density	Heterogeneous density (fatty tissue and soft tissue); may contain calcifications
Relationship to adjacent structures	Non-invasive	Infiltrates adjacent structures
Septa	Absent in most cases. If present, they are thin and sparse	Present. Abundant and thick
Time course	Stable	Rapid growth

Fourth is pancreatic teratoma. Its appearance is often variable depending on the type of tissue it contains; in some cases, when it contains a high amount of fatty tissue, it can be mistaken for a lipoma. However, they are typically heterogeneous, with cystic elements and a solid component whose density differs from that of fat. It may contain coarse calcifications. It is most commonly located in the head [2,5].

Regarding management, most patients do not require treatment, as this is usually an incidental finding; in these cases, a watch-and-wait approach is recommended, with periodic imaging follow-up to assess any changes in the size of the lesion. However, in the presence of complications, such as obstruction of the bile duct, pancreatic duct, or adjacent vascular structures, or when suspicion of malignancy persists despite imaging findings, surgical management may be indicated. The options described include pancreatoduodenectomy, enucleation, or distal pancreatectomy, depending on the location and characteristics of the lesion [7,8].

## Conclusions

Pancreatic lipoma is a rare benign neoplastic entity that is usually detected incidentally in most cases. It primarily affects middle-aged and older adults, with no gender preference, and is mainly located in the head or uncinate process of the gland. Its diagnosis relies on contrast-enhanced abdominal CT, which allows for the identification of the characteristic fatty attenuation of the lesion. The radiologist plays a crucial role in distinguishing this condition from malignant diseases such as liposarcoma; and in the presence of atypical findings, MRI is extremely useful, as they allow for the identification of benign characteristics specific to lipomas, such as hyperintensity on T1- and T2-weighted sequences and signal suppression on STIR and Fat-Sat sequences, which enable an accurate diagnosis even in large lesions. Adequate imaging characterization ensures conservative management in asymptomatic patients, reserving surgical intervention exclusively for those cases with symptoms associated with mass effect or compression of adjacent structures.
